# Draft Genome Sequence of the Commensal *Escherichia coli* Strain F-18

**DOI:** 10.1128/genomeA.01416-16

**Published:** 2016-12-22

**Authors:** Michael J. Ormsby, Síle A. Johnson, Daniel M. Wall

**Affiliations:** Institute of Infection, Immunity and Inflammation, College of Medical, Veterinary and Life Sciences, University of Glasgow, Glasgow, United Kingdom

## Abstract

Here, we report the draft genome sequence of *Escherichia coli* strain F-18, originally isolated from the feces of a healthy individual in 1977. The draft genome is 5,246,829 bp, with a G+C content of 50.50%, and it encodes 4,933 predicted coding sequences (CDSs), 10 rRNAs, and 84 tRNAs.

## GENOME ANNOUNCEMENT

*Escherichia coli* F-18 is a clinical strain that was originally isolated in the United States in 1977 from the feces of a healthy individual (1). F-18 contains seven plasmids, is of serotype rough:K1:H5, produces colicin V, and makes type 1 pili. This strain has also been demonstrated to be an excellent colonizer of the streptomycin-treated mouse large intestine (2, 3) and has been used in *in vivo* studies (4). As *E. coli* F-18 was recovered from a healthy individual, this genome sequence will thus serve as a useful resource for future studies into human intestinal pathogens, as a comparison to pathogenic strains.

Genomic DNA of *E. coli* F-18 was extracted from a freshly grown single colony using an Illumina Nextera XT DNA sample kit, as per the manufacturer’s protocol (Illumina, San Diego, CA). Sequencing was performed by Illumina MiSeq using a 2 × 250 paired-end protocol. Read quality analysis and trimming were conducted using Trimmomatic (5) and the quality assessed using in-house scripts combined with SAMtools (6), BedTools, and BWA-MEM (7). *De novo* assembly was conducted with SPAdes version 3.5 (8), resulting in a total of 101 contigs, with 84 contigs larger than 1,000 bp. The draft genome of *E. coli* F-18 is 5,246,829 bp, with 50.50% G+C content, and it encodes 4,933 coding sequences (CDSs), 10 rRNAs, and 84 tRNA sequences. Bioinformatic prediction of antimicrobial resistance genes using ResFinder (version 2.1) (9) revealed only the sulfonamide resistance gene *sul2*. VirulenceFinder (version 1.5) (10) identified seven genes with potential roles in virulence, including in serum survival and as siderophore receptors.

### Accession number(s).

This draft genome project has been deposited at DDBJ/EMBL/GenBank under the accession number MLZI00000000 (BioProject PRJNA348710; BioSample SAMN05914511). The version described in this paper is version MLZI01000000.
